# Normal Infant Immunologic Assessment and Uneventful Live Rotavirus Vaccination Despite Continuous Tofacitinib Exposure In Utero and During Breastfeeding

**DOI:** 10.1093/crocol/otae006

**Published:** 2024-01-20

**Authors:** Kenneth Ernest-Suarez, Luis E Murguía-Favela, Kerri L Novak, Remo Panaccione, Cora Constantinescu, Cynthia H Seow

**Affiliations:** Division of Gastroenterology and Hepatology, Department of Medicine, University of Calgary, Calgary, Alberta, Canada; Section of Hematology/Immunology, Department of Pediatrics, University of Calgary, Calgary, Alberta, Canada; Division of Gastroenterology and Hepatology, Department of Medicine, University of Calgary, Calgary, Alberta, Canada; Division of Gastroenterology and Hepatology, Department of Medicine, University of Calgary, Calgary, Alberta, Canada; Section of Infectious Diseases, Department of Pediatrics, University of Calgary, Calgary, Alberta, Canada; Division of Gastroenterology and Hepatology, Departments of Medicine and Community Health Sciences, University of Calgary, Calgary, Alberta, Canada

**Keywords:** inflammatory bowel disease, ulcerative colitis, tofacitinib, JAK inhibitor, breastfeeding, pregnancy, immunophenotyping

## Abstract

**Background:**

Janus kinase (JAK) inhibitors are effective for the treatment of inflammatory bowel disease (IBD). However, this class of medications is not recommended during pregnancy or breastfeeding based on animal data suggesting teratogenesis and recent reports of transmammary transfer after maternal ingestion, raising concerns for immune system development in babies exposed to these drugs.

**Methods:**

We present the case of a patient with IBD treated with a JAK inhibitor who decided to continue the medication throughout her pregnancy and during breastfeeding. This is the first reported case of a detailed immunologic profile in a baby exposed to tofacitinib in utero and during lactation.

**Results:**

A 30-year-old female with ulcerative colitis with previous exposure to vedolizumab and infliximab achieved complete remission with tofacitinib therapy. The patient became pregnant after 5 months of JAK inhibitor therapy and decided to continue tofacitinib during pregnancy and while breastfeeding. The patient delivered a healthy offspring with no congenital malformations, a normal detailed immunologic profile, and subsequent safe provision of the live oral rotavirus vaccine.

**Conclusions:**

This case highlights the importance of individualized counseling for patients of childbearing age who are candidates for JAK inhibition. Those who are pregnant or breastfeeding with refractory disease may have limited medical therapeutic options. Ongoing effective therapy for IBD resulted in complete disease remission in the mother and favorable outcomes in the infant. Further, an in-depth infant immunological assessment can lead to specific vaccination recommendations in exposed infants.

## Graphical Abstract

**Graphical Abstract:**
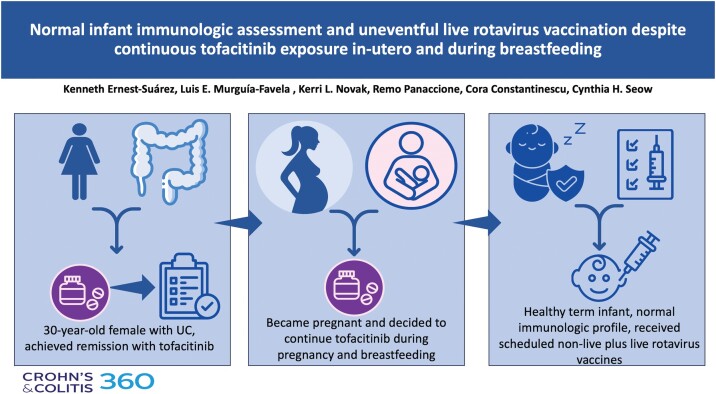

## Introduction

Janus kinase (JAK) inhibitors are effective induction and maintenance agents for the treatment of immune-mediated disorders and have become part of the armamentarium for treating inflammatory bowel disease (IBD).^1^ However, they are currently not recommended for use during pregnancy based on animal data demonstrating teratogenicity, albeit at doses far exceeding that used in humans.^2^

Further, it has been recently reported that tofacitinib can be detected in human breast milk up to 14 hours after maternal ingestion. This underscores the current recommendation of avoiding breastfeeding in the presence of JAK inhibition, given the theoretical impact on immune system development in infants, potential implications for vaccine responses, and risk of infection.^3^ As such, there may be hesitancy to prescribe JAK inhibitors in female patients of childbearing age who may wish to get pregnant.

## Case Presentation

Informed consent was obtained from the patient for this case report as per our institutional ethics board guidelines (Ethics ID: REB 15-1871). We report the use of tofacitinib during pregnancy and breastfeeding in a 30-year-old female with a 4-year history of ulcerative pancolitis with concomitant diagnoses of pyoderma gangrenosum and erythema nodosum. Previous medication exposure included mesalamine, prednisone, vedolizumab, and infliximab, with the development of paradoxical psoriasis with the latter. She was treated with tofacitinib 10 mg twice daily and achieved clinical, biochemical, sonographic, and endoscopic remission.

Preconception counseling had been provided regarding the limited data of tofacitinib effects on human pregnancies, and concerns regarding teratogenesis from animal data were reiterated.^2^ Five months into therapy, the patient conceived. Despite counseling to stop the JAK inhibitor, given the warnings surrounding pregnancy and lactation, she continued therapy but opted to de-escalate to 5 mg twice daily at 23-week gestation. She remained in steroid-free remission for the remainder of the pregnancy.

She subsequently developed pregnancy-induced hypertension, prompting induction of labor at term (37 weeks). Delivery was complicated by the need for an emergency Cesarean section due to arrested labor. The pregnancy resulted in an infant weighing 3.37 kg at birth with no congenital anomalies and no need for neonatal intensive care. After delivery, the patient chose to breastfeed despite existing recommendations and advice from the IBD pregnancy clinic to consider tofacitinib cessation or alternate feeding.^2^ There were no clinical or biochemical signs of disease reactivation during the post-partum.

The infant was seen at 12 weeks at the Special Immunization Clinic of a tertiary referral hospital, where infants exposed to biologic therapies are routinely assessed.^4^ Of note, there has been no reported data on immunologic assessment of infants indirectly exposed to JAK inhibitors.

An in-depth immunological assessment of the infant revealed a normal complete blood count with only mild thrombocytosis; normal IgG, IgA, IgM, and IgE serum levels; and overall normal extended T-cell and B-cell immunophenotyping by flow cytometry. There were nonclinically significant reductions of some memory T- and B-cell subsets. The infant had normal levels of T-cell receptor excision circles (TRECs) on newborn screening, correlating with a normal count of Recent Thymic Emigrants at the complete immunological assessment at 12 weeks of age (Table 1). In light of this normal immunologic assessment, Pediatric Infectious Disease recommended the infant proceed with the live oral rotavirus vaccination and the 2-dose schedule Rotarix vaccine (GlaxoSmithKline, Mississauga, ON, Canada) was provided at weeks 13 and 20. No adverse events following immunization, including severe vomiting/diarrhea or intussusception, were reported with active (chart review to 12 months of age) and passive (mandated public health reporting) surveillance. Mother and infant remain well up to 12 months follow-up.

**Table 1.** Immunological assessment at 12 weeks of age.

Laboratory measure	Result, absolute counts (10^9^ cells/L)	Reference ranges for infants aged 60–149 days, absolute counts (10^9^ cells/L)
Blood cell counts^a^		
White blood count (leukocytes)	10.6	5.0–19.5
Platelet count	**492 (H)**	150–400
Neutrophils	2.7	1.0–9.0
Lymphocytes	6.5	2.5–16.5
Monocytes	0.8	0.5–1.8
Eosinophils	0.4	0.2–0.6
T lymphocyte subsets^b^		
Total T cells (CD3+)	4.160	2.2–9.2
Helper T cells (CD3+CD4+)	3.173	1.6–6.5
Cytotoxic T cells (CD3+CD8+)	0.740	0.3–3.4
Recent thymic emigrants (CD3 + 4+45RA+31+)	2.385	1.4–5.2
Regulatory T cells (CD3 + 4+25 + 127-)	0.212	0.12–0.53
Naïve helper T cells (CD3 + 4+45RA+27+)	3.138	1.6–6.0
Naïve cytotoxic T cells (CD3 + 8+45RA+197 + 27+)	0.610	0.29–1.65
Terminally differentiated helper T cells (CD3 + 4+45RA+27-)	0.012	0.0-0.01
Terminally differentiated cytotoxic T cells (CD3 + 8+45RA+197-27-)	0.015	0.013–0.82
Central memory helper T cells (CD3 + 4+45RA-27+)	**0.000 (L)**	0.003–2.2
Central memory cytotoxic T cells (CD3 + 8+45RA-197 + 27+)	**0.001 (L)**	0.002–0.19
Effector memory helper T cells (CD3 + 4+45RA-27-)	0.001	0.001–0.021
Effector memory cytotoxic T cells (CD3 + 8 + 45RA-197-27-)	**0.001 (L)**	0.002–0.4
B lymphocyte subsets^b^		
Total B cells (CD19+)	0.742	0.52–2.3
Naïve B cells (CD19+CD27-IgD-)	0.648	0.62–2.12
Transitional B cells (CD19+IgM++CD38++)	0.229	0.13–0.94
Total memory B cells (CD19 + 27+)	**0.026 (L)**	0.040–0.23
Memory nonswitched B cells (CD19 + 27+IgM+IgD+CD38dimCD24+)	0.25	0.20–0.2
Memory class switched B cells (CD19 + 27+IgM-IgD-CD38dimCD24-)	**0.002 (L)**	0.010–0.17
Memory IgM + only B cells (CD19 + 27+IgM+IgD-)	0.002	0.0003–0.021
Plasmablasts (CD19 + 27++IgD-IgM-CD38++)	0.004	0.000-0.040
Immunoglobulin levels^a^	g/L	g/L
IgG	2.78	2.60-14.0
IgA	0.07	0.00-1.20
IgM	0.37	0.14-1.40
IgE	0.2	0.0-117

Abnormal results in bold.

H, High; L, Low, as per reference ranges.

^a^Reference normal values offered by Canadian laboratories.

^b^Reference normal values for lymphocyte immunophenotyping by flow cytometry are a combination of up-to-date published values^5^ plus internal validation with age-matched healthy controls at the Flow Cytometry Laboratory, Alberta Precision Laboratories, Calgary, AB, Canada.

## Discussion

The favorable outcomes of this pregnancy in a highly refractory ulcerative colitis patient exposed to a JAK inhibitor are consistent with the 11 pregnancies with maternal exposure to tofacitinib in the OCTAVE program, where no neonatal deaths, fetal deaths, or congenital malformations were documented.^6^ Although detectable tofacitinib levels have been detected in human breast milk as reported in a case study,^3^ we have demonstrated that despite this infant’s exposure to ongoing tofacitinib in utero and during breastfeeding, no clinically significant adverse developmental or immunologic effects were observed.

It is known that tofacitinib inhibits signaling via cytokine receptors associated with JAK 1 and 3, and, to a lesser extent JAK 2. JAK 3 is expressed in hematopoietic cells and is essential for signal transduction via the common γ-chain; thus, it is necessary for activation, differentiation, and homeostasis of lymphocytes by reducing STAT-1 and STAT-3 phosphorylation. Immunologic assessment of the infant was deemed necessary given that lymphopenia, neutropenia, and anemia have been reported with JAK inhibition.^7^ Further, in vivo and in vitro studies have demonstrated that tofacitinib can impair the differentiation of CD4+ helper T cells and strongly impair plasmablast development from naïve B cells, which may impact immunoglobulin secretion.^8,9^ No significant alterations were evident on this baby’s immunological assessment while breastfeeding at 12 weeks of age.

This research adds to the growing evidence surrounding the use of JAK inhibitors in young women. It highlights the need for individualized assessment in women of childbearing age who are candidates for JAK inhibition, including those who are pregnant or breastfeeding who may not have other therapeutic options apart from surgery.^2^ It is paramount to involve maternal–fetal medicine in these discussions and to ensure appropriate pregnancy monitoring. An in-depth infant immunological assessment and involvement of Pediatric Infectious Disease specialists can aid in infant vaccination recommendations. In this case, the normal immunologic assessment enabled the provision of all routine non-live infant vaccinations up to 12 months of age, and in addition, the live oral rotavirus vaccine, which has proved highly effective in reducing hospitalizations and emergency department visits for acute gastroenteric infant illnesses.^10^ Ultimately, the attainment of ongoing disease remission during pregnancy and breastfeeding with highly effective therapy, in this case, tofacitinib, in a patient with previous refractory disease, led to favorable maternal and infant outcomes.

## Data Availability

Data are not publicly available.
